# Effectiveness of peri-conception low dose of a highly gluconeogenic fast-release supplement in a fixed-time insemination program on ovarian and reproductive outcomes in meat goats

**DOI:** 10.1590/1984-3143-AR2025-0061

**Published:** 2026-07-20

**Authors:** Caroline Pessoa da Silva, César Carneiro Linhares Fernandes, Alfredo José Herrera Conde, Camila Muniz Cavalcanti, Felipe Brener Bezerra de Oliveira, Saul Gaudencio, Juliana Paula Martins Alves, Dárcio Ítalo Alves Teixeira, Davide Rondina

**Affiliations:** 1 Faculdade de Veterinária, Universidade Estadual do Ceará, Fortaleza, CE, Brasil; 2 Faculdade de Veterinária, Universidade de Fortaleza, Fortaleza, CE, Brasil; 3 Instituto Federal do Amapá – IFAP, Porto Grande, AP, Brasil

**Keywords:** FTAI, glycerin, follicle, corpus luteum, pregnancy

## Abstract

This study verified the impact of glycerin supplementation during fixed-time insemination on ovarian and reproductive outcomes in meat goats. Fifty-one Anglo-Nubian crossbred does underwent estrus synchronization with 12 days of CIDR, followed by 0.075 mg of d-cloprostenol and 150 IU of eCG. Thirty-six hours after CIDR removal, 100 μg of GnRH was administered, and 18 h later, insemination (AI) was performed via intrauterine laparoscopy. At CIDR insertion, goats were grouped into the Control Group (n = 25) fed a TMR, and the DGS Group (n = 26), where TMR was supplemented with 100 mL of glycerin from CIDR insertion to two weeks after AI. DGS showed a lower diet intake (-13%; P < 0.001) and higher (+ 5.3%; P = 0.026) glucose levels. In the CIDR interval, DGS pregnant goats (DGSPG), presented in the first wave a higher number of growing follicles (P = 0.035) and greater follicular diameters (P < 0.05). After CIDR removal, the DGSPG exhibited a lesser small follicles (P = 0.016), greater follicle size (P = 0.005), and lower number of corpora lutea (P = 0.031). Pregnancy (32.0% vs. 46.2%) and twinning rates (87.5% vs. 66.7%) were similar in Control and DGS groups (P > 0.05). The control group had a higher proportion of non-pregnant goats that were follicular unresponsive during the CIDR interval (P < 0.01). In pregnant animals, no differences (P > 0.05) were found in fetal biometric measurements, kidding rate, or litter size. Glycerin stimulated follicle growth and CL regression before AI but did not promote reproductive effects post-AI.

## Introduction

Artificial insemination (AI) is an assisted reproductive technique that allows genetic improvements in ruminant production systems. However, the most efficient use of this method in ruminants has been made possible through its association with hormonal protocols that enable the control of luteal and follicular functions and facilitate the implementation of fixed-time AI (FTAI) (Baruselli et al., 2018a, b). Currently, in different countries, FTAI is the most widely used protocol to support AI in cattle (Bó et al., 2019), sheep (Silveira et al., 2021), and goats (Arrebola et al., 2022). In Brazil in 2024, 91.8% of AI procedures were performed using FTAI (Baruselli, 2025). With the use of FTAI, it is possible to inseminate cyclic or anestrus females, induce the return of cyclicity in females during postpartum anestrus, and eliminate the need to detect estrus before AI (Rocha et al., 2022).

FTAI in dairy and beef cattle in South America is most frequently performed with estradiol-based hormonal protocols in the form of estradiol benzoate and progesterone-releasing devices (P4). In North America and Europe, the most widely used protocol in beef cattle is based on GnRH, called the Co-Synch protocol (Bó et al., 2019). In South America, however, this protocol is primarily used in dairy cattle (Bó et al., 2018). In small ruminants, available results have shown that it is possible to use FTAI successfully. In these species, the use of long-term protocols has been common, lasting 12–14 d in sheep (Kleemann et al., 2024) and 11–17 d in goats (Gore et al., 2020), a period based on the half-life of the corpus luteum (CL) (Ramos and Silva, 2018). Sheep and goats also receive an intravaginal pessary that releases synthetic progesterone analogs such as medroxyprogesterone acetate (MAP) and fluorogestone acetate (FAP), or an intravaginal device containing natural progesterone such as reduced-size CIDR (Ramos and Silva, 2018; Arrebola et al., 2022).

Despite considerable progress made in recent years regarding the efficiency of FTAI protocols in ruminants, AI is still primarily used in intensive dairy goat production systems (Luo et al., 2019). In meat goats, reproductive control technologies are used relatively rarely because of the extensive management systems commonly used. Especially in arid and semiarid regions, such as the Brazilian Northeast, where the main breeding farms of this species are concentrated, the predominant production systems rely on pasture-based feeding under harsh environmental conditions, characterized by seasonal fluctuations in forage availability. In these regions, nutritional control of herds is the main challenge in formulating rations, which is an essential prerequisite for implementing techniques such as FTAI. In this context, the use of supplements is a highly common path for technicians and breeders because it allows for diet control during specific and punctual periods of critical nutritional stress, such as the mating period. They can be modulated according to the nutritional status of the herd and allow the use of alternative nutritional resources, thereby reducing the pressure on cereals used in human food. In this sense, a large space is reserved for glucogenic precursors, such as glycerin, a product of the biodiesel chain that is easily accessible (ANP, 2024). The use of glycerin has some substantial advantages as an energy supplement in ruminant feed, such as the ease of administration directly in the diet or orally because it is an easy-to-handle product with a high energy density (Andrade et al., 2022). Glycerin serves as a rapidly available source of glucose. In goats, administration of 100–300 mL doses leads to a peak in plasma glucose levels 6 h after administration, with elevated levels lasting up to 24 hours. In contrast, peak insulinemia occurs 12 h after the dosing and lasts up to 24 h (Rodrigues et al., 2015).

However, in the case of glycerin, the time of supplementation and method of administration are factors that must be considered because they are directly involved in the response of the animal. In mice, high doses of glycerin cause an increase in circulating insulin levels and hyperglycemia, which have detrimental effects on oocyte competition and development, embryonic development, and pregnancy rates (Zhang et al., 2022). In ruminants, glycerin is added to the TMR (Andrade et al., 2022), concentrated (Fioravanti et al., 2024), or orally (Sotgiu et al., 2021; Silva and Silva, 2023). Andrade et al. (2022) observed a greater increase in ovulation rate in sheep when glycerin was administered by drenching or in TMR for 7 days before ovulation. The favorable effects of glycerin on ovarian activity were observed when glycerin was administered before ovulation in goats [200 mL for 7 days (Alves et al., 2019, 2021) or 6 days (Silva and Silva, 2023)], and in sheep [200 ml for 6 days (Oliveira et al., 2017), 150 ml for 7 days (Andrade et al., 2022) or 100 ml for 4 days (Sotgiu et al., 2021)].

Despite these results, the use of glycerin in FTAI is particularly challenging because of the complexity of the reproductive events involved, such as follicular growth, ovulation, fertilization, and embryonic development, each of which is characterized by different environments and requirements. The nutritional conditions required for follicular development and ovulation may differ from those required for subsequent embryonic development; however, these two events are closely interconnected. Souza et al. (2008) obtained high-quality embryos using exogenous insulin before or after mating in goats, which did not occur when stimulation was continuous with propylene glycol. Rodrigues et al. (2015) observed a reduction in pregnancy rate in animals supplemented with 300 mL of glycerin before mating. In small ruminants, periconceptional nutritional interventions aimed at maximizing reproductive performance require at least a few weeks for completion during mating. This type of management is traditionally used to increase the ovulatory response but is often associated with unfertilized ovulations or embryos that stop developing (Kakar et al., 2005). The capacity of an oocyte to successfully develop into an embryo is acquired during the capacitation process preceding ovulation. During the interval between fertilization and the maternal-embryonic transition stage, when transcriptional activity is initiated, embryonic function is supported by maternal RNA, and proteins synthesized during oocyte maturation can be influenced by nutrition (Pisani et al., 2008). Glucose transporters are essential for post-implantation embryonic development This indicates that poor nutritional management can alter RNA and protein synthesis several weeks before use.

Based on these assumptions, we hypothesized that the inclusion of low doses of glycerin in the diet of meat goats as a gluconeogenic precursor with high and rapid release in the periconception period would allow for better efficiency of the FTAI protocol by stimulating the ovarian response and pregnancy rate in the animal. Therefore, the present study aimed to investigate the impact of glycerin administration during the fixed-time insemination protocol and post-AI in meat goats and to verify the follicular dynamics, luteal activity, pregnancy rate, and early fetal development in these animals.

## Methods

### Location, animals, pre-experimental period and feeding management

The study was conducted at the farm of the School of Veterinary Medicine at Ceará State University, Brazil located at 4º2’23” S and 38º38’14” W from the school farm flock. All procedures were approved by the Ethics Committee for Animal Experimentation of Ceará State University (number 38257569/2016). Goats were homogeneous in body weight, subcutaneous sternal fat thickness (Table 1), ages (41.7 ± 9.6 months; overall mean ± SD), and body condition score (2.7 ± 0.2, from 1 to 5) and were kept in open collective shed stalls with free access to mineral supplements and water. Adipose mass was estimated by ultrasonographic measurement of the thickness of the subcutaneous fat deposits at the third sternebrae (Teixeira et al., 2008), due to its close relationship with the nutritional condition in goats (Hervieu et al., 1991).

**Table 1:** Body weight, fat sternal subcutaneous thickness prior the FTAI protocol. Feed intake and peripheral glucose level during FTAI protocol in goats fed with maintenance diet (Control group) or supplemented with glucogenic precursor (DGS group).

**Parameters**	**Group**			**p Value**	**Time**	**G x T**
	**Control**	**DGS**	**SEM**	**Group**		
*Carcass marker and body weight*						
SSFT*, mm	11.2	10.3	0.343	0.194	-	-
Initial BW*, kg	32.4	32.3	0.958	0.953	-	-
Fasting BW**, kg	31.1	31.0	1.021	0.968	-	-
*Feed Intake*						
DMI, g/kg MW	59.1	51.4	0.546	< 0.001	0.009	0.904
DMI, % BW	2.5	2.2	0.023	< 0.001	0.002	0.874
*Metabolite plasma*						
Glucose, mg/dL	51.0	53.7	0.588	0.026	0.183	0.029

Prior to the experiment, the animals were subjected to 30 days of adaptation and housed in a common pen, where health and reproductive controls were performed according to Fernandes et al. (2018b). Briefly, endo- and ectoparasite treatments were administered and the subjects were vaccinated against clostridiosis. Cyclicity and ovarian function were monitored by ultrasound and sexual receptivity of the fertile mature buck. Goats received the same diet, composed of a TMR based on chopped elephant grass and concentrate. A feed mixture of TMR was prepared in water and furnished to satisfy the nutritional requirements for maintenance in adult non-dairy goats (NRC, 2007). The diet was provided in two daily meals at 08h00 and 15h00, and feed intake was monitored daily during the experimental period.

### Fixed-time artificial insemination (FTAI) procedures

#### Estrus and follicular wave synchronization

**Figure 1:**
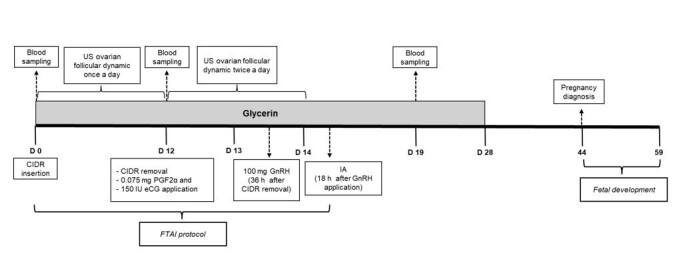
General experimental design including dietary treatment and hormonal protocol for laparoscopic artificial insemination.

The goats had their estrus and follicular wave synchronized (Figure 1) by insertion 14 days before artificial insemination (AI) of an intravaginal device impregnated with progesterone (CIDR®, Controlled intravaginal drug release; InterAg, Hamilton, New Zealand). After 12 days, the device was removed and 0.075 mg of d-cloprostenol (Prolise®; ARSA S.R.L., Buenos Aires, Argentina) and 150 IU of eCG (Folligon®; Intervet Canada Inc., West Hill, Ontario, Canada) were applied. Thirty-six hours after removal of the CIDR, 100 μg of GnRH (Gestran-plus®, a.i. lecirelin; ARSA S.R.L.) was administered. AI was performed 18 h after GnRH (Biehl et al., 2017).

#### Intrauterine laparoscopy AI

Intrauterine laparoscopic AI was performed according to Menchaca and Rubianes (2004) using fresh semen collected through an artificial vagina from two Anglo-nubian bucks with previously proven fertility through the analysis of sperm parameters (color, volume, appearance, motility, vigor, and concentration). The insemination dose was diluted in a TRIS-yolk-based extender to a sperm concentration of 50 × 10^6^. On the day before insemination, does were fasted for 24 h and watered for 12 h. The anesthetic protocol used was a combination of acepromazine 1% (0.1 mg/kg) (Acepran® 1%, Vetnil, São Paulo, Brazil), xylazine 2% (0.1 mg/kg) (Anasedan® 2%, Ceva, São Paulo, Brazil) and ketamine 10% (2 mg/kg) (Dopalen® 10%, Ceva, São Paulo, Brazil).

### Experimental design

At CIDR insertion, the goats were randomly grouped in two diet-treatment (Figure 1): the DGS Group (n = 26), in which the TMR feed mixture was prepared with a solution of 100 mL of glycerin (99.9% glycerol) and water in a 9:1 ratio, and the Control Group (n = 25) where was maintained the initial TMR diet.

Glycerin solution was included daily in the TMR for 4 weeks, from CIDR insertion to two weeks after the AI laparoscopy procedure. According to the energy density of glycerol, 0.38 Mcal of EM/moL (Mach et al., 2009), the energy value of 100 mL of glycerol was estimated as 0.51 Mcal of EM, which represents an increase of 39.2% in the energy maintenance requirement (NRC, 2007) provided by TMR in Control Group.

### Ultrasonography analysis of ovarian follicular dynamics and luteal activity

Ultrasonography was performed once daily from CIDR insertion to CIDR removal (Figure 1) and twice daily from CIDR removal to the AI procedure. Ovarian images were obtained with B-mode ultrasound equipment using a 5 MHz linear transrectal probe (model Z5 Vet; Mindray Bio‐Medical Electronics Co., Shenzhen, China). Images were captured and analyzed using ImageJ software (National Institutes of Health, Millersville, MA, USA), which was previously calibrated, the mean diameter of each identified follicle and corpus luteum (CL) was determined as the average between its vertical and horizontal dimensions (Conde et al., 2023). Follicles observed in both ovaries were categorized based on size into small (< 3 mm), medium (≥ 3 and < 5 mm), and large (≥ 5 mm) (Ginther and Kot, 1994).

### Pregnancy diagnosis and early fetal development

Pregnancy was diagnosed 45 days after AI (Figure 1). According to the methodology described by Del'Aguila-Silva et al. (2021), fetal development was evaluated with goats in a standing position, using a B-mode real-time transabdominal ultrasound with a 5 MHz convex transducer (model Z5 Vet; Mindray Bio‐Medical Electronics Co., Shenzhen, China). The probe was positioned in the right inguinal region of the animal or immediately in front of the udder, with the urinary bladder as the main reference structure for identification of the reproductive tract. The diameter of the embryonic vesicle and crown-rump length were measured at days 30 and 45 of gestation (Figure 1). Biparietal, abdominal, and thoracic diameters on day 45 of gestation.

### Glucose assay

Blood samples were taken from all animals at day of CIDR insertion, at CIDR removal and 7 days after, during glycerin supplementation (Figure 1), by jugular venipuncture into heparinized vacuum tubes (BD Vacutainer®, Franklin Lakes, NJ, USA), performed before the morning feed is provided. The samples will then be centrifuged at 3000 rpm for 15 min to separate the plasma, from which three aliquots will be obtained and stored at -20 °C. Analyses of glucose, were performed by spectrophotometry in automated biochemical analyzer (Mindray® BS 120, Mindray Biomedical Electronics Co., Shenzhen, China) using commercial kit (Bioclin®, Quibasa, Minas Gerais, Brazil), according to the manufacturer’s instructions. The sensitivity of the assay for glucose was 1.31 mg/dL and the intra- and inter-assay CV were 2.59% and 0.78%, respectively, as indicated by the manufacturer.

### Statistical analysis

Statistical analyses were performed using Statistica Software version 13.4.0.14 (2018; TIBCO Software, Inc., Palo Alto, CA, USA). The data were initially verified for mathematical assumptions using the Shapiro-Wilk test. If these conditions were not met, log10x transformation was applied. Data were analyzed using the General Linear Model (GLM) procedure for analysis of variance. The factors included in the model were group (Control, DGS), interval of the sample assessment (time), and interaction group vs. time. All pairwise comparisons were performed using the Newman–Keuls post hoc test. For the pregnancy rate, twin rate, pregnancy failure rate, mortality rate, and kidding rate, the effect of the group was analyzed using the chi-square test. Correlation analysis between pregnancy diagnosis and body weight, sternal subcutaneous fat thickness, and follicle traits before and after CIDR was performed using Spearman’s test. Statistical significance was considered at the 5% level (p < 0.05).

## Results

### Body weight, feed intakes and peripheral glucose levels in supplementation interval

There was no significant difference in fasting body weight between the two groups prior to the laparoscopic procedure (P = 0.968) (Table 1). Animals supplemented with glycerin showed lower dry matter intake (-13%; P < 0.001) than the control group (Table 1). The DGS group also showed a significant increase (P = 0.026) in plasma glucose concentration compared with the control group (Table 1). A significant interaction between the group and time effects was observed (Interaction G × T, P = 0.029) due to the increase in glycemia in the DGS group (+3%) and a corresponding decrease in the control group (−11%) after CIDR removal. In the sample taken seven days later, peripheral glucose levels were 54.5 ± 1.2 mg/dL vs. 47.7 ± 1.7 mg/dL (P = 0.007) for the DGS and control groups, respectively. The highest blood glucose value recorded in the DGS group during supplementation was 69.3 mg/dL on the day of CIDR withdrawal.

### Follicles turnover throughout the FTAI procedure

#### Follicles turnover before CIDR removal

**Figure 2:**
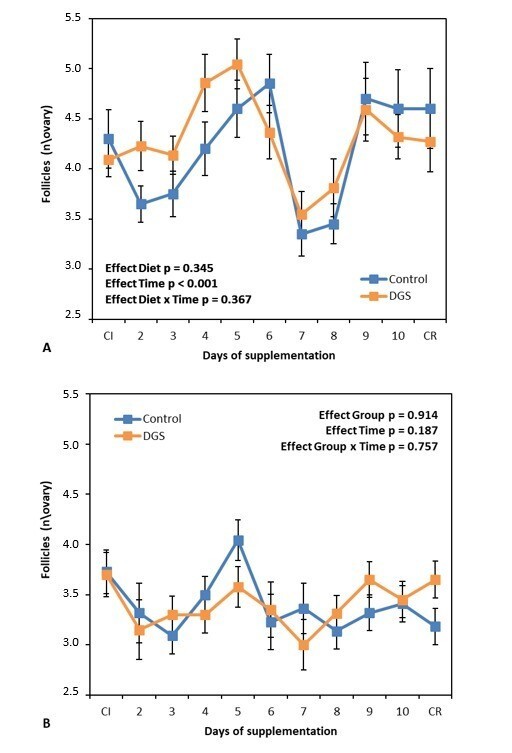
Total follicles counted by ultrasonography performed after CIDR insertion during FTAI protocol, in goats fed with maintenance diet (Control) or supplemented with glucogenic precursor (DGS), for pregnant (Figure 2A) and no-pregnant (Figure 2B) does, diagnosed by ultrasound at 45^th^ days after AI. In the figures CI and CR are respectively the day of CIDR insertion and the day of CIDR removal. Data are plotted as mean ± SEM. The P-value for the ANOVA effects for group, supplementation period (effect time) and interaction, are shown in figures. *P < 0.05.

Table 2 shows the follicular dynamics measured using ultrasound analysis during the FTAI protocol. There was a significant increase in the number of follicles as the CIDR interval progressed (Effect of Time, P < 0.001). There was also a difference in follicular traits in relation to pregnancy outcomes. Goats that became pregnant after AI presented a higher number of follicles and greater follicular size (P < 0.001) than non-pregnant animals. Thus, for a clearer presentation in Figure 2, we divided the follicular dynamics into two pregnancy groups. Table 2 details the follicle tracts in pregnant goats, as shown in Figure 2A.

**Table 2:** Follicles turnover in goats during FTAI protocol, fed with maintenance diet (Control group) or supplemented with glucogenic precursor (DGS group).

**Parameters**	**Group**			**p Value**	**Time**	**G x T**
	**Control**	**DGS**	**SEM**	**Group**		
*Follicles traits before CIDR removal*						
*Total follicles 1st day, n/ovary*	4.0	3.9	0.200	0.725	-	-
Total follicles, n\ovary	3.8	3.9	0.042	0.227	< 0.001	0.960
Follicle size, mm	3.1	3.1	0.030	0.433	0.215	0.177
*Follicles traits before CIDR removal, in pregnant goat after AI*						
Follicle size of 1th wave emergence day, mm	3.0	3.4	0.112	0.045	-	-
Largest follicle size of 1th wave, mm	4.7	5.3	0.131	0.036	0.030	-
Follicles ≥ 3 mm, n\ovary	1.7	2.1	0.097	0.035	0.045	0.440
Total follicles, n\ovary	4.2	4.7	0.108	0.017	0.002	0.854
Follicle size of 2th wave emergence day, mm	3.3	3.2	0.124	0.753	-	-
Largest follicle size of 2th wave, mm	5.2	5.0	0.177	0.625	0.256	0.078
Follicles ≥ 3 mm, n\ovary	1.9	1.8	0.120	0.570	0.098	0.297
Total follicles, n\ovary	4.1	4.2	0.157	0.675	0.001	0.434
*Follicles traits after CIDR removal*						
Follicles < 3 mm, n\ovary	2.1	2.0	0.063	0.163	0.001	0.152
Follicles ≥ 3 mm, n\ovary	1.8	1.9	0.043	0.481	< 0.001	0.328
Follicle size 48 hours BL*, mm	3.4	4.0	0.141	0.011	-	-
Largest follicle size 18 hours BL, mm	7.2	7.6	0.151	0.135	-	-

* BL, before laparoscopy; Time, ANOVA effect for interval sampled used.

In pregnant animals from both the nutritional groups, two follicular waves were observed during the CIDR period (Figure 2A). In the first wave, on days 3–6 of supplementation, an increase in the follicular number and size was observed in both groups (Effect of Time, P < 0.05; Table 2). The DGS group had a higher number of follicles (P = 0.017) and growing follicles (P = 0.035) than the control group (Table 2). The first wave ended on days 5 and 6 in the DGS and control groups, respectively. The follicular diameter at the beginning of the wave and its maximum point was higher (P < 0.05) in DGS goats (Table 2). On day 7 of CIDR (Figure 2A), after one week of supplementation, the second wave began in both groups (Effect of Time, P < 0.001), which recorded the highest number of follicles two days later, on day 9 of supplementation. In the second wave, no differences (P > 0.05) were recorded between the groups in terms of the number of follicles and follicular diameters on the day of emergence or the largest size (Table 2). In non-pregnant animals in both groups, the number of follicles (Figure 2B) was significantly lower (P < 0.001) than in pregnant animals (4.2 ± 0.06 n/ovary vs. 3.4 ± 0.05 n/ovary). The follicular diameter was also significantly lower (P < 0.001) than in pregnant animals (3.2 ± 0.04 mm vs. 3.0 ± 0.05).

#### Follicles turnover after CIDR removal

After the withdrawal of CIDR and subsequent application of eCG, on the 12th day of supplementation, there was a significant increase (Effect of Time, P < 0.001; Table 2) characterized by an increase in the number of growing follicles (>3 mm) and a reduction in the number of small follicles (P = 0.001) in both groups. In this last follicular class, a significant lower number of follicles in pregnant goats in the glycerin group was also observed compared to the control group (2.4 ± 0.1 n/ovary vs. 1.9 ± 0.2 n/ovary; P = 0.016). This difference did not occur in non-pregnant animals (1.9 ± 0.1 n/ovary vs. 2.0 ± 0.1 n/ovary; P = 0.298).

Regarding follicular diameter, the measurement performed 48 hours before laparoscopy (Table 2), at the time of eCG application, also recorded a larger diameter in the glycerin group compared to the control group. However, this difference occurred in pregnant animals (4.3 ± 0.3 mm vs. 3.0 ± 0.2 mm; P = 0.005), and did not verify in non-pregnant animals (3.7 ± 0.3 mm vs. 3.8 ± 0.2 mm; P = 0.815). The follicular diameter measured before laparoscopy did not differ between the groups (Table 2).

### Luteal activity throughout the FTAI procedure

#### Luteal activity before CIDR removal

Regarding luteal activity (Table 3), the DGS group animals, there was a lower number of CL in relation to the control (P < 0.001) but recording a larger diameter (P < 0.05). For non-pregnant animals, both groups showed a lower number and diameter than pregnant animals (P < 0.001), and similar between them, respectively 0.2 ± 0.02 n/ovary vs. 0.1 ± 0.01 n/ovary (P = 0.100) for the number of CL and 5.7 ± 0.2 mm vs. 5.4 ± 0.3 mm (P = 0.378) for the luteal diameter.

**Table 3:** *Corpus Luteum (CL) in* goats during FTAI protocol, fed with maintenance diet (Control group) or supplemented with glucogenic precursor (DGS group).

**Parameters**	**Group**			**p Value**		**G x T**
	**Control**	**DGS**	**SEM**	**Group**	**Time**	
*CL after CIDR insertion*						
CL, n\ovary	0.3	0.2	0.011	< 0.001	0.756	0.991
CL diameter, mm	5.7	6.5	0.127	0.036	0.398	0.873
*CL after CIDR removal*						
CL, n\ovary	0.1	0.05	0.132	0.031	< 0.001	0.994
CL diameter, mm	6.4	5.5	0.235	0.067	0.040	0.974

Time, ANOVA effect for interval sampled used.

#### Luteal activity after CIDR removal

After CIDR removal (Table 3), there was a significant reduction in the proportion of CL per ovary (effect time, P < 0.001) and in luteal size (effect time, P = 0.037) in both groups. The DGS group presented a lower number of CL/ovary compared to the control group (P = 0.031), and there was no significant difference (P > 0.05) in luteal diameter between the treatments.

Pregnant animals showed a lower proportion of CL compared to those negative to DG (0.05 ± 0.02 n/ovary vs. 0.1 ± 0.02 n/ovary; P = 0.010).

### Reproductive outcome after AI and fetus development in pregnant goats

Table 4 shows the reproductive outcomes after artificial insemination. There was no significant difference (P > 0.05) between the nutritional groups in terms of pregnancy and twin rates, with overall mean values of 43.1% and 68.4%, respectively. Pregnancy loss was concentrated in both groups during the interval between AI and the pregnancy diagnosis performed 45 days after AI (Table 4). Only one animal in the glycerin group aborted after this period (Table 4). In the control group, animals that experienced pregnancy loss were significantly more likely to have fewer follicles during the CIDR interval (P < 0.01; Table 4). In the same group, positive and statistically significant correlations (Table 5) were found between pregnancy outcome and the number of growing follicles (0.69; P = 0.001) and number of CL (0.59; P = 0.007), measured before CIDR removal. No correlations were found between pregnancy, live weight, subcutaneous sternal fat thickness, number of growing follicles, and CL after CIDR removal (Table 5).

**Table 4:** Reproductive outcome after AI in goats fed with maintenance diet (Control group) or supplemented with glucogenic precursor (DGS group).

**Parameters**	**Group**			**p Value**
	**Control**	**DGS**	**SEM**	**Group**
*Pregnancy outcome*				
Pregnancy rate, % (n/n)	32.0 (8/25)	46.2 (12/26)	-	0.779
Twinning rate, % (n/n)	87.5 (7/8)	66.7 (8/12)	-	0.828
Pregnancy failure*, % (n/n)	68.0 (17/25)	53.8 (14/26)	-	0.392
Follicular unresponsive BCR**	82.4 (14/17) A	71.4 (10/14)	-	-
Follicular responsive BCR	17.6 (3/17) B	28.6 (4/14)	-	-
Mortality***, % (n/n)	-	8.3 (1/12)	-	-
*Delivery outcome*				
SSFT, mm	13.0	11.8	0.352	0.096
BW, kg	41.0	40.3	1.802	0.869
Kidding rate, % (n/n)	100.0 (8/8)	91.7 (11/12)	-	0.862
Twinning rate, % (n/n)	75.0 (6/8)	72.7 (8/11)	-	0.755
Litter size, n (n/n)	1.9 (15/8)	2.0 (22/11)	0.178	0.903

* Gestation failure from AI to pregnancy diagnosis; **BCR: before CIDR removal; ***Mortality from the pregnancy diagnosis to delivery. BW, body weight; SSFT, subcutaneous sternal fat thickness. A, B P < 0.01, between rows in the same column.

**Table 5:** Correlation coefficients between pregnancy outcome, body weight, subcutaneous sternal fat thickness, follicle traits, and corpus luteum proportion before and after CIDR in goats fed with maintenance diet (Control group) or supplemented with glucogenic precursor (DGS group).

**Parameters**	**Group**			
	**Control (n = 25)**	**p Value**	**DGS (n = 26)**	**p Value**
Initial BW before FTAI procedure	0.21	0.304	0.45	0.064
SSFT, mm	0.36	0.069	0.29	0.153
*Before CIDR removal*				
Follicles ≥ 3 mm, n\ovary	0.69	0.001	0.45	0.061
CL, n/ovary	0.59	0.007	-0.03	0.902
*After CIDR removal*				
Follicles ≥ 3 mm, n\ovary	0.28	0.245	0.01	0.968
CL, n/ovary	0.28	0.249	-0.29	0.234

BW, body weight; SSFT, subcutaneous sternal fat thickness.

In pregnant animals, no differences in fetal measurements were observed between the groups (Table 6). The diameter of the embryonic vesicle and the craniocaudal length of the fetus increased in both groups with gestation time (Time Effect, P < 0.001).

**Table 6:** Ultrasonography early fetometric measurements in pregnant goats after AI, fed with maintenance diet (Control group) or supplemented with glucogenic precursor (DGS group).

**Parameters**	**Day**	**Group**			**p Value**	**Time**	**G x T**
		**Control**	**DGS**	**SEM**	**Group**		
Diameter of embryonic vesicle*, mm	30-45	45.8	43.3	1.443	0.226	< 0.001	0.333
Crown-rump length*, mm	30-45	30.9	29.6	1.947	0.611	< 0.001	0.906
Biparietal diameter, mm	45	13.4	13.1	0.974	0.949	-	-
Abdominal diameter, mm	45	1.1	1.3	1.204	0.666	-	-
Thoracic diameter, mm	45	1.2	1.1	0.202	0.643	-	-

* Time, ANOVA effect for interval of measurement used.

At parturition (Table 4), no differences were observed between the treatments in terms of live weight and sternal subcutaneous fat thickness, nor did the diving rate, twin rate, and litter size differ between the groups (P > 0.05). These parameters have mean values ​​of 86%, 74%, and 2.0, respectively.

## Discussion

The evidence collected in this study partially confirmed the initial hypothesis regarding the impact of the inclusion of a low dose of glycerin as a glucogenic precursor in the diet of goats during the periconception period of the FTAI protocol. From a reproductive perspective, the selected supplementation strategy induced a more favorable scenario in terms of follicular turnover during the permanence of the progestin-releasing device and its subsequent removal before ovulation. In contrast, the extending glycerin supplementation post-AI did not enhance the benefits observed in follicular growth, which did not lead to improvements in pregnancy rate and fetal development.

However, the use of glycerin in the diet at the chosen dosages effectively increased the peripheral glycemic concentrations in the experimental animals, which also resulted in a lower diet intake. Both phenomena were expected and did not reach critical values ​​for animal efficiency. The maximum blood glucose value recorded in the supplemented group was within the range observed in goats (Bezerra et al., 2023; Das et al., 2024). No clinical signs of hyperglycemia were observed, whereas dry matter intake remained within the recommended values ​​of nutritional requirements (NRC, 2007) for the weight class (> 2.2% BW). Transient changes in diet consumption in animals supplemented with glycerin are frequently recorded (Andrade et al., 2024; Fioravanti et al., 2024) and are consequences of the homeotropic response of the animal to the increased energy density of the diet, as well as to the transient increase in ruminal propionate released during glycerin degradation. Postprandial fatty acid concentrations are recognized modulators of feed intake in ruminants (Fioravanti et al., 2024).

The glycemic increase allowed the supplemented animals to successfully modulate follicular dynamics before and after the presence of CIDR and to present a larger CL diameter. After the administration of eCG preceding AI, the DGS group showed high follicular depletion during the induction of the follicular wave, a larger follicular size, and a more efficient induced regression of the CL. This was evidenced by the substantial reduction in the presence of CL compared to the control group after the withdrawal of CIDR and successive application of prostaglandin.

Several studies have reported the positive effects of glycerin supplementation on ovarian activity in sheep (Sotgiu et al., 2021; Andrade et al., 2022) and goats (Alves et al., 2021; Silva et al., 2023). Follicular growth and ovulation rates are favored when increased circulating glucose promotes increased concentrations of insulin and insulin-like growth factor (IGF-1), which act directly on the hypothalamic-pituitary-ovary axis (Guo et al., 2019; Sotgiu et al., 2021). The development of follicular waves depends on the steroidogenic function of the ovaries (Rawan et al., 2023). The steroidogenic function of granulosa cells (GC) is regulated by glucose metabolism through metabolic pathways such as phosphatidylinositol 3-kinase, protein kinase B (PI3K-PKB/Akt) and AMP-activated protein kinase (AMPK) (Mo et al., 2024). Estradiol, which promotes follicular growth and aromatase activity to convert androgens into estrogens (Rawan et al., 2023), is produced by GC through the intervention of glycolytic metabolites such as the enzyme pyruvate kinase M2 (PKM2), steroidogenic acute regulatory protein (STAR), 3β-hydroxysteroid dehydrogenase (3β-HSD), cytochrome P450 aromatase (P450AROM) and cytochrome P450 cholesterol (P450SCC) (Wang et al., 2022). The use of glycogenic or energy diets also promotes the activity of the metabolic hormone IGF-1 (Kaewlamun et al., 2020; Rahbar et al., 2021). This hormone is involved in the synthesis of factors such as progesterone (P4), androstenedione, activin A, inhibin A, and follistatin (Piau et al., 2023), which promote follicular waves (Adams and Singh, 2021). Furthermore, IGF-1 promotes follicular growth because of its ability to act as a co-gonadotropin (Hayes et al., 2024) and acts synergistically with follicle-stimulating hormone (FSH) to stimulate GC proliferation and estradiol production (Piau et al., 2023; Baddela et al., 2025).

The results shows that extending supplementation in the post-AI period did not provide positive advantages for the success of the FTAI protocol in terms of a higher pregnancy rate, prolificity, or fetal development. However, the pregnancy rates observed in the present study were similar to those reported by Holtz et al. (2008), Stelletta et al. (2017), and Fernandes et al. (2018a) in goats subjected to FTAI. Similarly, the kidding rate was comparable to or higher than those reported in other studies conducted on goats, by Holtz et al. (2008), Hashemi and Safdarian (2018), Fernandes et al. (2018a), Gore et al. (2020), Arrebola et al. (2022) and Wondim et al. (2022).

Dietary supplementation during the peri-conception period is an established nutritional strategy frequently used in small ruminants to induce an appropriate environment for events associated with oocyte maturation and competence, fertilization, implantation, and embryonic development (Kakar et al., 2005). Although these events are strongly associated with better results using the FTAI protocol (Vasconcelos et al., 2018), in the present study, no advantages were observed in terms of the number of pregnancies or multiple pregnancies. In addition to parturition, both groups exhibited similar reproductive responses.

In our study, the evaluation of pregnancy losses indicated that most of these in both groups occurred prior to AI and were concentrated in animals that did not show significant follicular growth in the period preceding CIDR removal. In the non-supplemented animals, the relationship between pregnancy and ovarian response before CIDR removal was more evident. In this group, a significant proportion of nonpregnant animals presented with low follicular growth during the CIDR period. In these animals, pregnancy was also significantly and positively correlated with the number of growing follicles and CL present during the same period. In contrast, in the supplemented group, the inclusion of glycerin in the diet seemed to alleviate this phenomenon. In these animals, better follicular response and CL quality were recorded during the CIDR period. Furthermore, the lower proportion of CL after CIDR withdrawal suggests that the induction of luteal regression was much more efficient in these animals than in the control group.

The presence of a functional CL at the beginning of a FTAI protocol is usually associated with an increase in pregnancy (Murugavel et al., 2003). However, effective luteolytic induction is essential for a new follicular wave to successfully reach ovulation. The larger the follicle, the greater the chance of achieving pregnancy. According to Vasconcelos et al. (2018), the best results in FTAI are essentially guaranteed through four physiological events: initiation of a synchronized follicular wave, promotion of follicular growth and selection of a dominant follicle, decrease in the concentration of circulating progesterone through lysis of the corpus luteum, and induction of ovulation in synchrony with AI.

In FTAI, fertility rate is also associated with the protocol used. Martemucci and D’Alessandro (2011) indicated that the long-term FTAI protocol is associated with ovulation originating from aged follicles, which, in turn, can lead to a lower pregnancy rate. In these protocols, around the 6th day, the progestin device decreases the concentration of P4, maintaining it at subluteal levels (Menchaca and Rubianes, 2004), causing slower follicular renewal (Viñoles et al., 2001; Menchaca and Rubianes, 2002). Menchaca and Rubianes (2002) reported that in goats, the number of follicular waves depends on the concentration of circulating P4, and those that are exposed to lower concentrations of P4 consequently present lower follicular turnover. Subluteal concentrations of P4 increase LH pulses that promote follicular growth until the end of the hormonal protocol, causing ovulation of persistent dominant follicles, which are in the process of aging and compromise the fertility rate (Viñoles et al., 2001; Menchaca and Rubianes, 2004). Follicles with a longer lifespan compromise the quality of the oocyte, which ages at the time of ovulation (Evans et al., 2001; Viñoles et al., 2001). A favorable option is the application of short protocols using vaginal pessaries lasting 5 to 9 days. This protocol maintains P4 at adequate levels that favor the growth and ovulation of new follicles because of adequate suppression of the LH pulse frequency (Nakafeero et al., 2020), allowing better or similar conception rates to long-term protocols (Ramos and Silva, 2018; Gore et al., 2020; Nakafeero et al., 2020). The use of P4 is sufficient to induce estrus, which occurs two–three days after P4 treatment; however, gonadotropins are necessary to synchronize estrus and ovulation (Ramos and Silva, 2018). eCG, administered at doses of 250–1000 IU on the last day of P4 treatment, stimulates the onset of estrus 48 h later and ovulation approximately 60 h later (Ramos and Silva, 2018; Nakafeero et al., 2020). The administration of synthetic GnRH analogs between 12 and 36 h after removal of the vaginal pessary also allows for the induction, synchronization, and improvement of the ovulation rate, favoring the use of FTAI, as the success of the protocols depends on the synchronization between the moment of insemination and ovulation (Ramos and Silva, 2018; Arrebola et al., 2022).

The presence of low follicular responsiveness was partly expected, and occurred essentially due to the presence of animals with low follicular renewal. However, this phenomenon was not associated with anestrus in the present study. The selected animals were representative of the regional population and were characterized by continuous cyclicity throughout the year, likely due to the relatively stable photoperiod of the region. Nutritional effects were considered negligible, given the reproductive and nutritional control performed in the pre-experimental adaptation period and the lack of correlation between follicular parameters, the presence of the CL, and the live weight and adipose mass of the animal. However, the fact that it was manifested in both groups, although at different intensities, suggests the involvement of specific tracts in the animals used in the experiment. Meat goats originate from extensive farming, are more nervous than dairy animals, are handled less frequently, and may respond differently to excessive manipulation.

Animals with fewer follicles usually exhibit lower follicular renewal (Burns et al., 2005). In cattle with a low number of follicles, follicular dynamics are altered during the follicular selection phase because the granulosa cells of the follicles produce lower concentrations of E2, resulting in high concentrations of FSH that promote the constant development of intermediate follicles. In cows with a large number of follicles, E2 is released at higher concentrations, which suppresses FSH secretion and stimulates LH secretion, allowing follicular selection to continue with follicular dominance and subsequent ovulation (Sakaguchi et al., 2019). In a study conducted with goats, Kharayat et al. (2024) reported that a lower presence of antral follicles is associated with reduced concentrations of anti-Müllerian hormone, which, in turn, shows a positive correlation with reproductive parameters such as the number of ovulations per estrous cycle, ovarian surface area, and litter size.

## Conclusion

The inclusion of glycerin in the diet of meat goats two weeks before ovulation in the FTAI protocol allowed favorable alterations in follicular development. However, the extension of this supplementation to two weeks after AI did not offer substantial advantages in terms of pregnancy rate and prolificacy. The study also confirmed that reproductive performance in FTAI is highly dependent on the initial ovarian response of the animal, and that this can be positively supported by the presence of a rapidly available gluconeogenic inducer, such as glycerin. In view of this, we hope to conduct further investigations with the aim of improving the efficiency of this type of supplementation in FTAI protocols for goats for better efficiency and dissemination of this reproductive technology in herds.

## Data Availability

The data presented in this study are available upon request from the corresponding author due to legal reasons.
